# The mineralogy and structure of use-wear polish on chert

**DOI:** 10.1038/s41598-020-78490-0

**Published:** 2020-12-09

**Authors:** Patrick Schmidt, Alice Rodriguez, Kaushik Yanamandra, Rakesh K. Behera, Radu Iovita

**Affiliations:** 1 Department of Early Prehistory and Quaternary Ecology, Eberhard Karls University of Tübingen, Tübingen, Germany; 2 Applied Mineralogy, Department of Geosciences, Eberhard Karls University of Tübingen, Tübingen, Germany; 3 Anthrotopography Laboratory, Center for the Study of Human Origins, Department of Anthropology, New York University, New York, NY USA; 4 Composite Materials and Mechanics Laboratory, Mechanical and Aerospace Engineering Department, New York University, Tandon School of Engineering, Brooklyn, NY USA

**Keywords:** Archaeology, Mineralogy, Materials science

## Abstract

Polished edges of archaeological stone tools are commonly investigated to obtain information on the tools’ uses in prehistory. Yet to this day, it remains unclear what exactly such polishes are and how they form. Answering these questions should allow the elaboration of new interpretative methods based on objective measurements. Two major competing hypotheses of polish formation have been proposed: abrasion and the formation of a thin amorphous film on the chert or flint surface. We employ reflectance infrared spectroscopy, a technique particularly sensitive to thin amorphous films, to investigate these two hypotheses. We found no added amorphous layer that would have formed upon friction against bone, antler, ivory or wood. Our observations suggest polish formation by abrasion, notwithstanding previous claims of added amorphous surface structures. This has implications for our understanding of the physical processes taking place during friction of chert and flint against different materials. Our results also open the possibility to propose new pathways for identifying different use-wear processes, based on the degree of abrasion.

## Introduction

Along with residue analysis, the study of microscopic use-wear is one of only two currently known methods for obtaining information on how a prehistoric stone tool was used. Despite significant advances in the study of stone tool use-wear^1, 2^, most interpretations of use from wear traces left by worked materials still rely on analogies between experimental and archaeologically documented phenomena, rather than on an accurate understanding of the processes underlying these phenomena^3^. Among the different use-wear traces, it is widely believed that polishes hold important information for identifying and distinguishing the materials that were worked with ancient stone tools^4–7^. Yet to this day, it remains unclear what exactly a polish is and how it forms. Settling this question is crucial for improving and speeding up archaeological interpretations by developing methods to identify and distinguish different worked materials.

There are two major competing hypotheses of polish formation (the ‘abrasive’ model and the ‘additive’ or ‘silica gel’ model), with several others incorporating aspects of both^2,3,8,9^. The ‘abrasive’ model^10–14^ states that what we visually perceive as polish on the edge of a stone tool is the result of physical abrasion of the stone surface by contact with the worked material, resulting in the smoothing of its natural roughness. Several other authors^9,11,15^ favour variants of the abrasive hypothesis that includes the grinding effect of dirt particles or microscopic fragments of the tool itself^16^. However, for the ‘additive’ or ‘silica gel’ model^17^, polish on the tool is instead formed as a deposit on the chert surface. The newly deposited layer, or thin film, is in this case assumed to consist of amorphous silica^18^. The rationale behind this assumption is that, even under fast growth conditions, the formation of nanometer-sized quartz crystals at ambient or slightly elevated temperatures still requires up to two days^19^, a time span largely exceeding the formation of use-wear polish. If a secondary layer of silica is formed on the chert surface, it must therefore be amorphous silica. In this case the formation of thin film can be expected to include a step of mobilisation of the chert’s silica (proposed mechanisms are dissolution in water^17,20^ and melting caused by friction^16^).

Optical reflection microscopy is one of the most common techniques used to examine polished tool surfaces. However, this technique does not allow to unambiguously decide between the two hypotheses. The reason for this is that both mechanisms, addition of a film and abrasion, may lead to reduced surface roughness with respect to the unmodified chert surface. Reduced roughness, in turn, increases the quantity of light reflected back to the microscope’s objective through specular reflection, reducing the magnitude of diffused reflection^21^. The perceived higher reflection coefficient (brighter appearance of the polished zone) would in both cases appear as high relief, making the polish optically stand out of the observed surface. Thus, abrasion and the addition of a silica film cannot easily be told apart with optical reflection microscopy. Several other microscopic and chemical methods have been used to decide between these hypotheses (for the most recent comprehensive review see^8^). Various authors have focused on sequential experiments, where a stone tool was intermittently used, cleaned and inspected, to verify with light and electron microscopy whether morphological features of the original surface were abraded or filled in. For example, Ollé and Vergès^8^ showed that observable depressions cannot be the result of corrosion, as proposed by Sala^22^, because they were already present in the chert surface before use. Yamada^14,23^ had already noted that such depressions are not filled with any material during use. Meeks et al.^13^ suggested, using SEM micrographs of sectioned sickle blades, that a visually unmodified chert structure is preserved all the way to the polished surface, concluding that there was no additional amorphous silica layer. Others attempted to study the surface with chemical analyses. For example, Plisson^24^ found that some polishes disappeared upon corrosive attack with Na_2_CO_3_, concluding that polish must be at least to some degree caused by a deposit (because amorphous silica, or any other added substance, has higher solubility in bases than quartz). Also proposing the addition of a film during polish formation, Christensen et al.^25^ artificially implanted copper ions into chert near its surface. They found that working did not remove the copper from the surface and concluded that use-wear polish must be formed by the filling of surface irregularities by the worked material. Unfortunately, the authors had not investigated the position of their copper ions in the chert surface or their diffusion into the chert, making conclusions on why copper is retained during working ambiguous. While the infilling of cavities was in contradiction with previous findings^14^, Šmit et al.^26,27^ built upon Christensen et al.’s theory^25^ and showed that elements coming from the worked material can be found on the chert surface after use (e.g., calcium from bone; phosphorus from wood). But Evans and Donahue^28^ showed that mild cleaning techniques, without an ultrasonic bath, could already lead to the misinterpretation of residues as being part of an additive layer of polish. Thus, the chemical evidence on polish formation does not appear to be conclusive but can be interpreted as pleading for an additive polish formation; interpretations based on microscopy rather point towards abrasion.

Using a more classical mineralogical approach, Masson et al.^12^ analysed the surface of sickle blades with X-ray diffraction (XRD). They detected no amorphous silica or opal-CT (an optically isotropic appearing interstratification of cristobalite and tridymite with low crystallinity^29^) at the surface and reported in favour of the abrasive hypothesis. While this may sound like conclusive evidence at first, it is important to highlight that amorphous silica produces only weak signals in XRD. Only a diffuse reflection centred around 23° *2θ* is caused by the short-range order of the structure. This amorphous dome is sometimes barely visible as background distortion. This also means that thin films of amorphous SiO_2_ may appear essentially transparent to X-rays, causing underlying crystalline structures (the unmodified chert) to still be apparent as discrete crystal reflections (the penetration depth of a standard Cu K-α ray in SiO_2_ is 20–100 µm, depending on the incident angle). Thus, XRD is not suited to investigate the deposition of thin layers of amorphous surface deposits. As it stands, none of the reported characterization techniques seem to unambiguously allow for deciding between the two hypotheses of use-wear polish formation. We therefore propose a more conclusive approach to investigate the potential formation of thin amorphous surface films.

**Figure 1:**
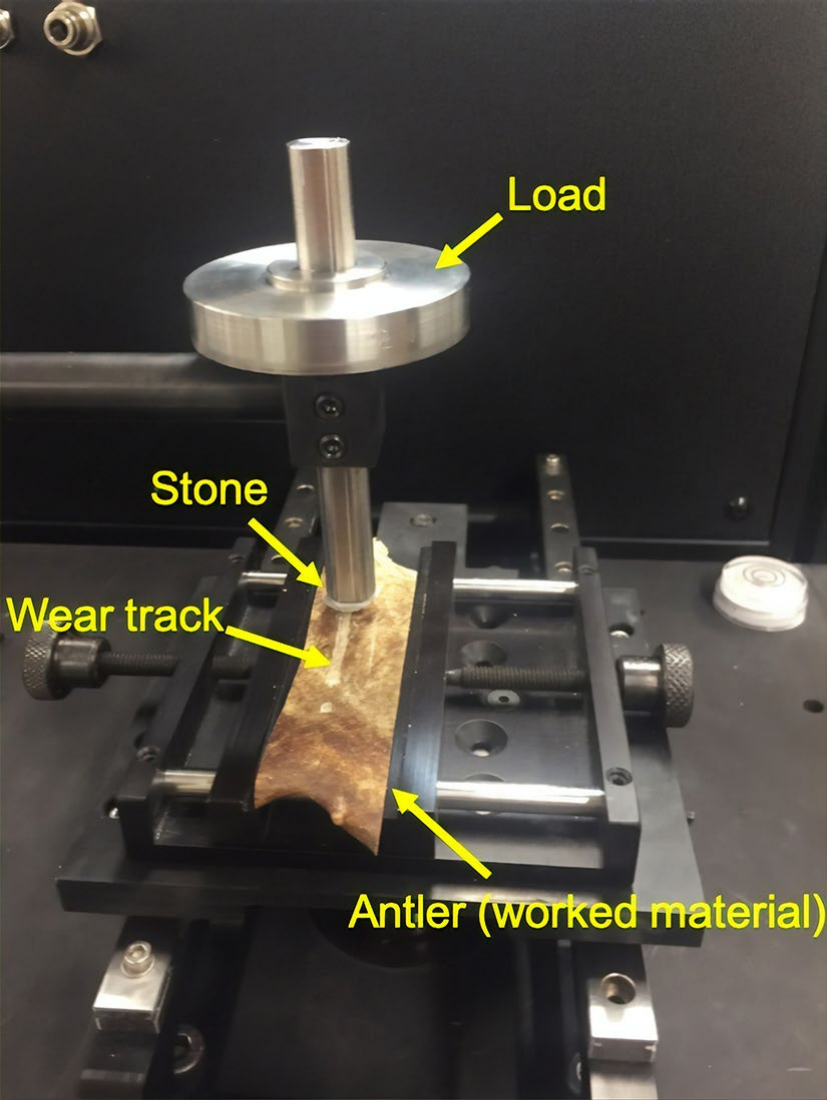
A typical tribology experimental setup highlighting antler as the worked material. The pin bearing the load (5 N shown here, 20 N used in the experiment) travels back and forth on the worked material to generate the wear track on the worked material and polish on the stone (chert). Photo by Kaushik Yanamandra.

In this study, we used reflection infrared (IR) spectroscopy to examine whether amorphous films form on the surface of chert during use. Reflection spectra are plots of reflection coefficient of IR radiation (reflectance) as a function of frequency, as given by the Fresnel equations (see for example:^30^), i.e. they are produced at the surface. Therefore, even the thinnest surface layers can be identified. If there is precipitation of dissolved silica onto the chert surface, that otherwise consists of quartz, reflectance spectroscopy should pick up a signal caused by the amorphous structure of the precipitate (the underlying quartz would not be visible). Using this technique, crystalline and amorphous silica can be distinguished as follows: the IR reflectance spectrum of quartz is different for polarisation planes parallel to the ordinary (O) ray and the extraordinary (E) ray (^30,31^). The O-ray spectrum shows a band near 800 cm^−1^ and the E-ray spectrum near 780 cm^−1^. Thus, the presence of both O- and E-ray bands in the unpolarised quartz spectrum is indicative of the mineral’s optical anisotropy. In the reflectance spectra of isotropic amorphous silica like opal-A (but also in the spectra of Opal-CT), there is a single band near 780 cm^−1^ (supplementary Fig. S1). The testing conditions for amorphous silica in use-wear polish are therefore: observing a single band near 780 cm^−1^ confirms the presence of a layer of opal-A at the surface of the polish. Observing a quartz spectrum with E- and O-ray bands shows that there is no amorphous phase present at the surface of use-wear polish. If only part of the surface contains an amorphous phase, the ratio between the reflectance values at 800 cm^−1^ and 780 cm^−1^ should be different between the polished and unpolished zones. To explore these testing conditions, we apply a protocol combining confocal and laser-scanning microscopic observations and reflectance IR spectroscopy. Infrared spectroscopy produces data on the mineralogy and crystalline structure of the polishes and laser-scanning microscopy is used for illustrative purposes. We investigate use-wear polish on chert produced on five target materials: antler, ivory, bone, and two types of wood (spruce and beech). These experiments are performed on a tribometer using the testing module to mimic linear reciprocating motion. The experimental set-up was customized to handle the samples of interest. One typical experimental set-up highlighting antler as the worked material is shown Fig. 1. We optimized the stroke length, load, and duration of contact to produce sufficiently large patches of polish to apply IR spectroscopic measurements.

## Results

### Infrared spectroscopy

**Figure 2:**
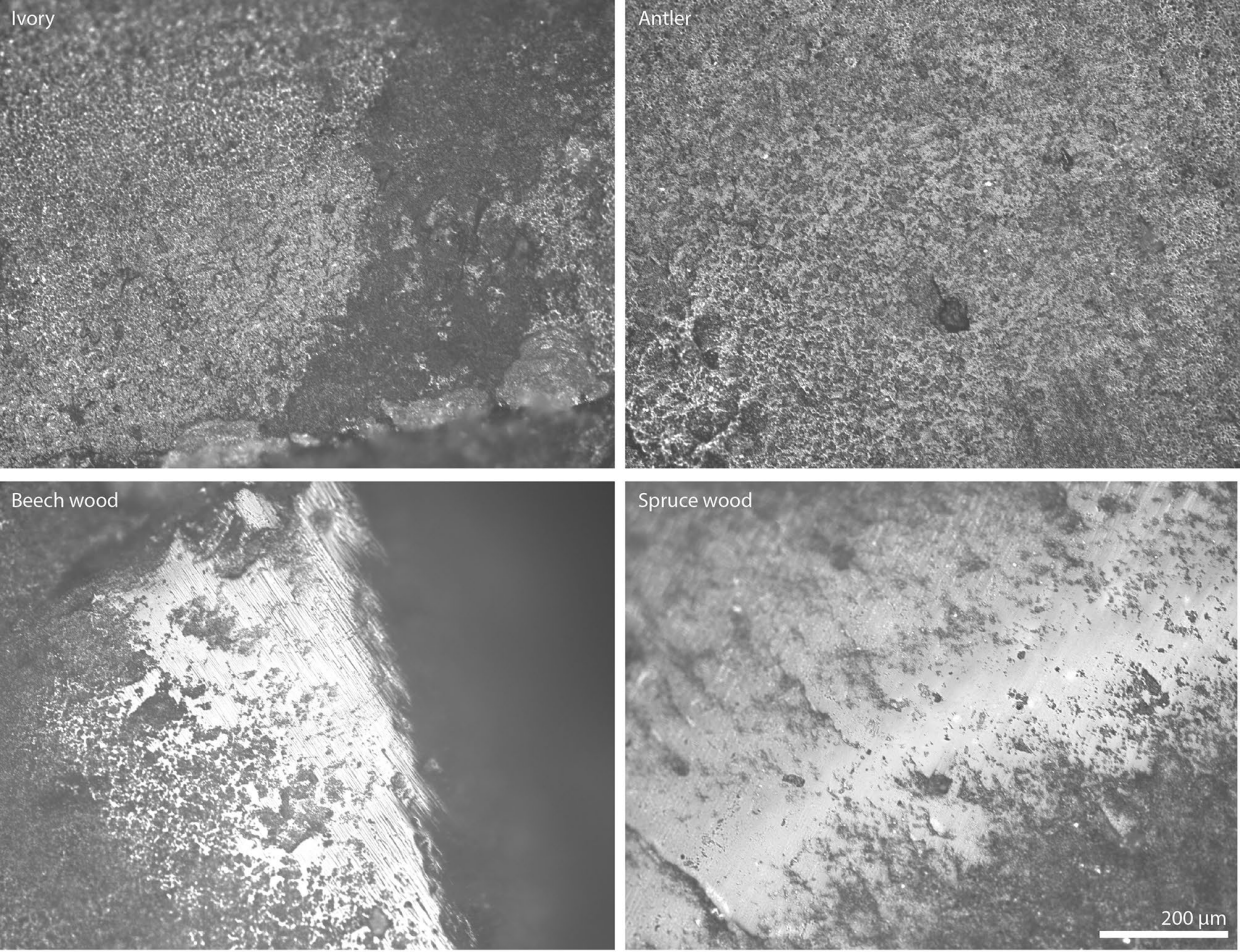
Reflection micrographs of use-wear polish on various samples analysed after the tribology.

**Figure 3:**
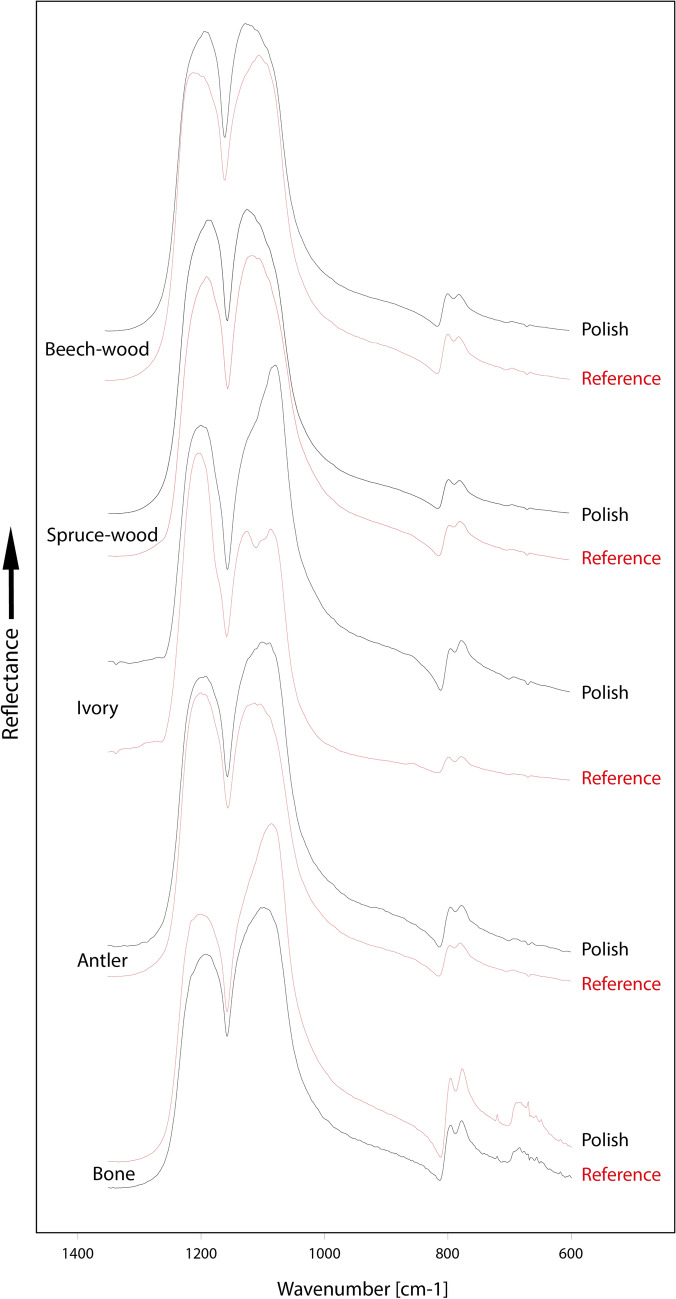
Reflection infrared spectra recorded on polished and unpolished zones of chert abrade against five target materials.

Examples of the polish as observed by reflection microscopy are shown in Fig. 2 and supplementary Fig. S2. Spectra of all samples are shown in Fig. 3. In all five samples, reflectance spectra recorded on use-wear polish are similar to reference spectra recorded on unused fracture surfaces. This is in terms of the Si–O stretching envelope near 1200 cm^−1^ and the spectral range between 815 and 700 cm^−1^, where the quartz O- and E-ray bands lie (i.e. none of the measurements made on a polished zone resulted in an amorphous spectrum). To investigate whether the quartz structure was subject to partial amorphisation in the polished zones or whether only parts of the polish contain amorphous silica, we compare the relative reflectance values at 779 cm^−1^ and 798 cm^−1^ between unused and polished zones (values in Table 1). In unused surfaces, the average ratio between the reflectance values of the five measurements at both wavenumbers is 1.05 with a variation of + 0.07 and − 0.12. Polishes on antler, beech wood, and bone samples fall within this range. Polish on the ivory sample produced a lower ratio than in the unpolished zone (i.e. the 798 cm^−1^ band is relatively higher in the polished spectrum) and the spruce wood polish produced a positive ratio value that falls outside of the 1.05 + 0.07 error range (i.e. the 779 cm^−1^ band is relatively higher in the polished spectrum). Thus, the IR-reflectance signature of the anisotropy of quartz remains unchanged between polished und unpolished zones in all but one sample. To verify these trends, the spectral zone between 815 and 700 cm^−1^ was fitted with two pseudo-Voigt functions (supplementary Fig. S3), to obtain both band components at 779 cm^−1^ and 798 cm^−1^. After deconvolution, the mean of all intensity ratios obtained from references spectra (unused surfaces) is 0.874 ± 0.11 (values in Table 1). In the spectra acquired on polished areas, again only the ivory and spruce-wood ratio values lie outside of this range. As for the measurement of absolute reflectance values, the direction of change in the ivory sample is towards greater anisotropy in the polished zone (i.e. the 798 cm^−1^ band is higher). The spruce wood sample produced a slightly higher 779 cm^−1^ band in the polished zone than in the unused zone. Ratios obtained from polished zones on antler, beech wood and bone samples fall within the measurement error of ± 0.11, thus confirming the trend observed before spectral fitting.

**Table 1:** IR Reflectance values and band components obtained by spectral deconvolution.

Target material	Measured on	Band position (cm^−1^)	Reflectance		Deconvolution		
			Intensity	Intensity ratio	Intensity	Width	Intensity ratio
Antler	Reference	779	0.159	1.05	0.087	20.95	0.91
		798	0.151		0.096	14.22	
	Polish	779	0.705	1.08	0.384	20.95	0.92
		798	0.655		0.419	14.22	
Beech wood	Reference	779	0.266	0.93	0.150	20.95	0.76
		798	0.286		0.197	14.22	
	Polish	779	0.373	0.97	0.212	20.95	0.80
		798	0.384		0.264	14.22	
Ivory	Reference	779	0.131	1.02	0.053	20.95	0.85
		798	0.128		0.063	14.22	
	Polish	779	0.469	1.21	0.234	20.95	1.09
		798	0.387		0.215	14.22	
Spruce wood	Reference	779	0.565	1.12	0.306	20.95	0.98
		798	0.506		0.311	14.22	
	Polish	779	0.364	0.97	0.208	20.95	0.82
		798	0.377		0.254	14.22	
Bone	Reference	779	13.303	1.11	2.641	20.95	0.86
		798	12.014		3.060	14.22	
	Polish	779	5.655	1.07	2.461	20.95	0.80
		798	5.279		3.060	14.22	

Values under “reflectance” are the absolute reflectance values at both wavenumbers. Values under “Deconvolution” were obtained by fitting the reflectance spectra.

### 3D surface models

**Figure 4:**
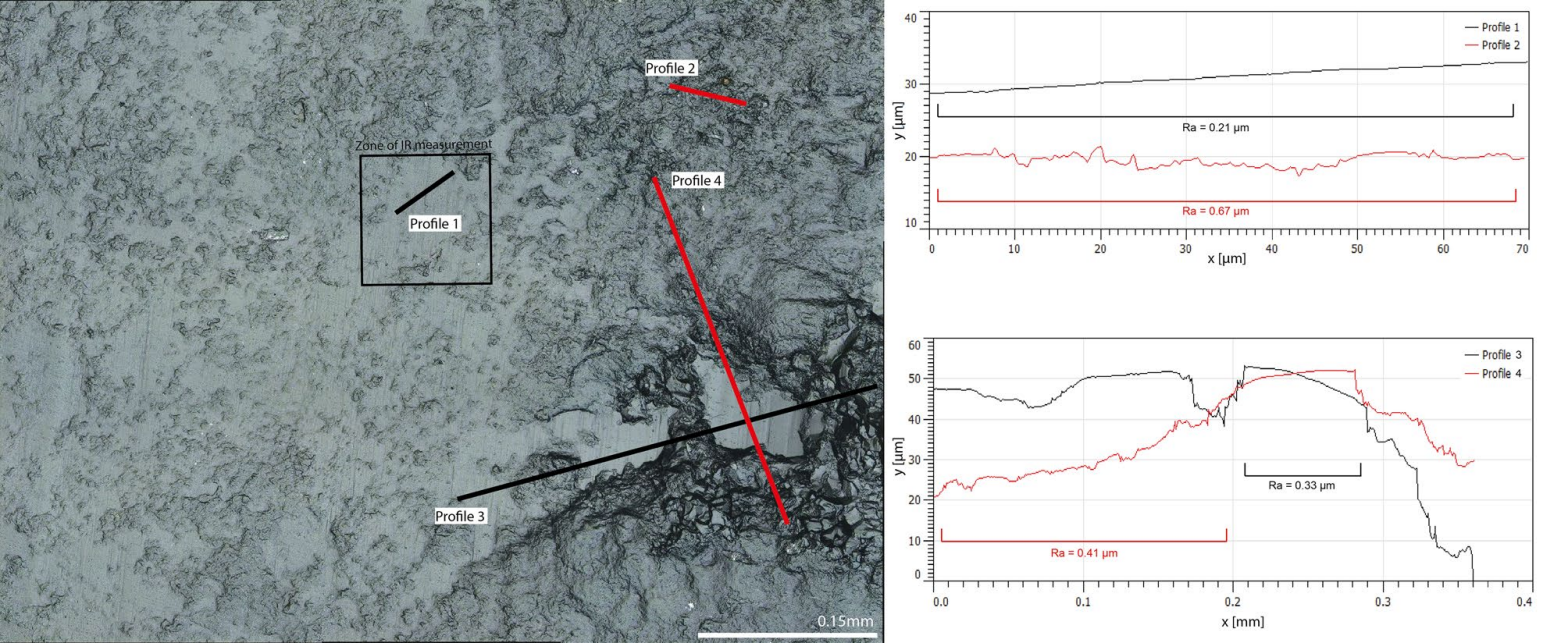
3D surface model of chert abraded against beech wood and the corresponding extracted profiles. Note the plateau effect created by the abrasion of the higher parts of the surface. Rendered with Gwyddion v. 2.4, http://gwyddion.net/^32^.

**Figure 5:**
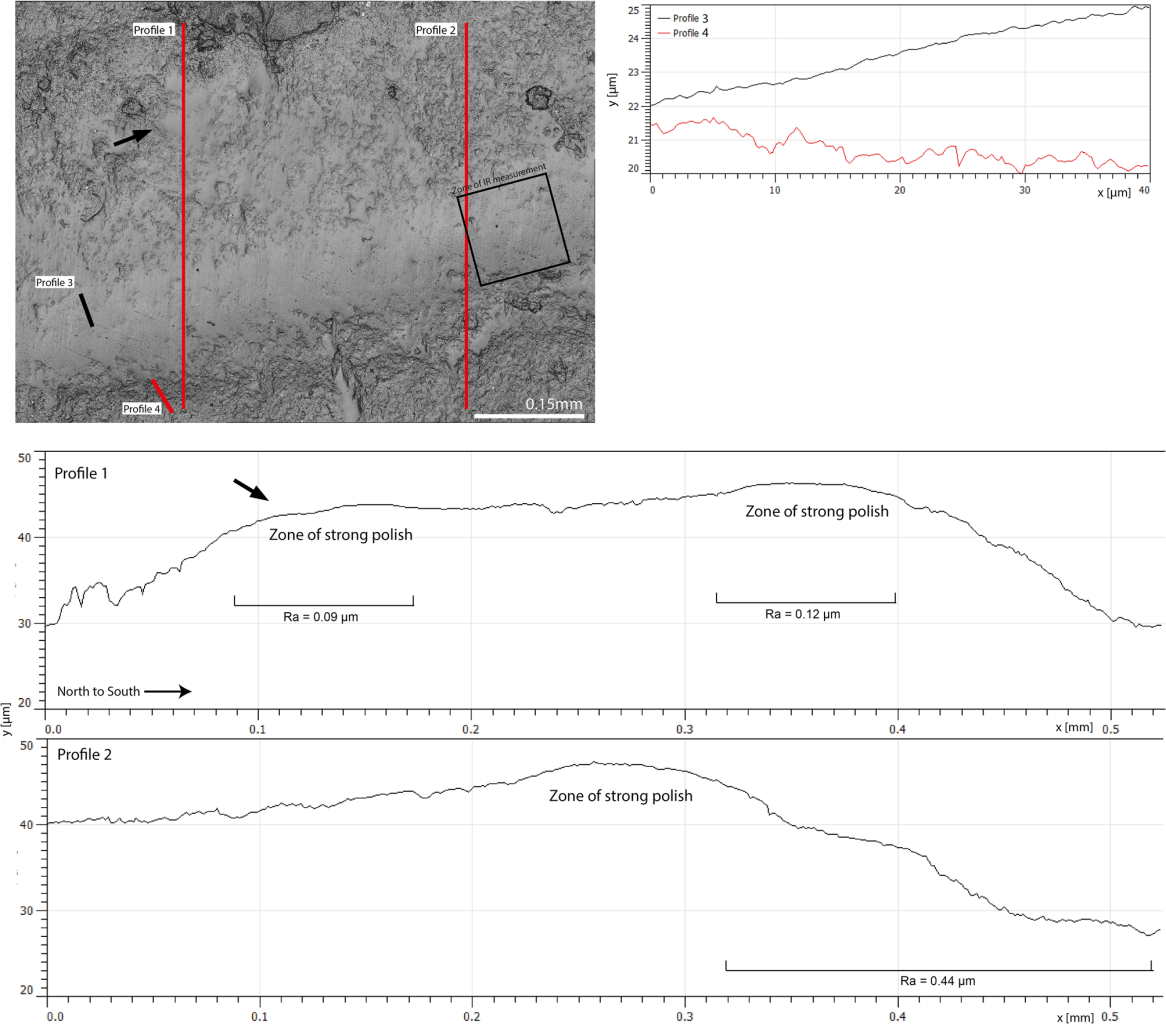
3D surface model of chert abraded against spruce wood and the corresponding extracted profiles. Note the stronger roughness in unpolished zones. Also note that the higher parts are more polished than the valleys. The zone indicated by a black arrow in the picture is the zone pointed out by the same arrow in profile 1. Rendered with Gwyddion v. 2.4., http://gwyddion.net/^32^.

3D surface models of the five used-wear polishes confirm the impressions gained from IR spectroscopy. In the two strongly polished samples (beech, Fig. 4, and spruce wood, Fig. 5) the polish only covers the highest parts of the surface relief. Higher parts on the fracture surfaces (like waves left behind by conchoidal fracture, see Fig. 2) are more affected by the polish than lower parts. Similarly, protruding zones are cut by the polish, leaving behind mesa-like plateaus (see profiles 3 and 4 in Fig. 4). These plateaus suggest that polishing involved the abrasion of structures that stick out most from the surfaces. In both wood samples, roughness average (Ra) values measured outside of the polished areas range from 0.4 to 0.7 µm. Ra measured on polished zones in both wood samples range between 0.1 and 0.2 µm. We note similar features in ivory and antler polish. There, polishes are weaker than in samples abraded against wood. Even in zones of relatively strong polish, a surface relief with an Ra of ~ 0.4–0.6 µm is present. More protruding zones of this surface relief are observed to be more polished than the lower zones. In ivory polish, a plateau effect (similar to but much weaker than the one seen in beech wood polish) flattens out the high parts of the surface relief (Supplementary Fig. S4). A similar trend can be observed in bone polish, although the lower signal to noise ratio partially masks this effect (Supplementary Fig. S5). While these observations alone do not preclude alternative interpretations of and additive process of polish to the highest parts of the chert reliefs, their combination with our infrared measurements (that has found no indication of such addition) allows for illustrating the surface topography of the abraded zones.

## Discussion

Our observations pinpoint that there is no layer of amorphous silica on the surface of the polished zones in all samples. All measured surfaces consist of quartz. Partial amorphisation or only small parts of the polished area covered by amorphous silica, which would cause an IR reflectance signal of lower optical anisotropy, can be ruled out in four of the five samples. Only polish on the spruce wood sample produced a signal that might be interpreted as leaning towards lower anisotropy. This result must, however, be considered in the light of the uncertainties that contributed to estimating the error range of the ratio value used to identify changes in anisotropy. The mean of the ratio between 779 and 798 cm^−1^ bands, and its measurement error, was calculated on five reference samples only. This merely provides an approximate idea of the measurement error.

In quartz single crystals, the relative intensity of both bands is a function of the angle of incidence of the IR radiation with respect to the quartz lattice axes (for changes in the relative intensity of O- and E-ray bands as a function of quartz crystal orientation see for example:^31^). Thus, most of the error is likely to be caused by crystal orientation. That our error ranges (+ 0.07 − 0.12, or ± 0.11) are most certainly too small, can be appreciated from the reflectance spectrum of ivory polish. Its ratio value falls outside of this error range, but in the direction of higher anisotropy. If this change were significant, it should be argued that ivory polish production corresponds to increasing anisotropy, but there are few crystallographic processes supporting such a conclusion (defect healing might be one of them). Thus, the error is likely to be greater than + 0.07 − 0.12 and ± 0.11 for absolute reflectance values and fitted band component respectively. Whether the spectrum acquired on spruce wood polish also falls into a hypothetical larger error range cannot be decided based on the available data but it appears likely. As it stands, polishes produced by the abrasion of chert against bone, antler, ivory, spruce wood, and beech wood do not differ in their mineralogy from unused chert surfaces. Sickle polish has in the past been studied because it is particularly strong^4,7,13,23,33^. For the automated experiments we conducted, however, it was not possible to build a set-up in which we could repeat linear motions on grasses. Therefore, our data only allow to make statements on the polishes obtained from contact with the analyzed five target materials. The question of sickle polish must therefore await future investigations.

A potential limitation to the interpretation of our spectroscopic data may come from an alternative interpretation of the nature of use-wear polish. Fullagar^15^ had performed experiments with ice made from distilled water and obtained polish. This made him believe that sub-microscopic chert particles could be infilling interstices of the chert structure. Such a mechanism was described not to require any amorphous cement from re-mobilized silica that would hold the polish together. Thus, such an addition would not be detected by reflection IR spectroscopy, as no amorphous phase would be present. Investigating this mechanism of polish formation by addition of sub-microscopic chert particles is beyond the scope of our study.

## Conclusion

Thus, our study concludes that no silica glass film is detected in polished zones of chert. Reflectance IR spectroscopy confirms that no supplementary amorphous layer is formed upon friction against bone, antler, ivory or wood. Further, in zones where polish appears on chert, material was removed from the fracture surface. This is supported by the plateau effect observed in our 3D surface models. For the reasons explained in the results and discussion, quartz neo-formation, in other words a crystalline film, cannot be expected to be sufficiently fast for it to play a role during use-induced polish formation. Our data therefore support polish formation through abrasion. This is in agreement with earlier hypotheses^10–14^, providing supplementary strong arguments against the formation of added films in polished zones. Establishing the abrasive model constitutes an important step for methodological development, especially for the use of quantitative roughness parameters to distinguish worked materials. Future studies should attempt to elucidate the mechanism of abrasion and how measurable physical properties of different worked materials affect the process.

## Materials and methods

### Samples and sample preparation

A single piece of Baltic/morainic flint from Denmark was used for all use-wear experiments described hereafter. Its texture is nano-crystalline non-oriented chalcedony. Infrared spectroscopy revealed no calcite impurities and the expected SiOH related defect structure was observed (as in^34^). These characteristics make representative of the large majority of upper cretaceous flint and many fine-grained cherts^35^. Four individual ~ 1 cm x ~ 1 cm x ~ 0.5 cm measuring slabs were cut from knapped flint flakes in a way that one of the two larger sides was a fracture surface. To obtain the intended ~ 1 × 1 cm size of the slabs, the flat pieces were snapped off after roughly sawing, so that at least one of the smaller sides of the slabs was also a fracture surface (i.e. not sawn). The flaked surface on the larger side was used for use-wear polish production during our tests. The fracture surface on the smaller side was used for reference measurements. To produce use-wear polish, flint slabs were mounted with epoxy resin onto cylindrical sample holders and abraded sequentially for 1, 3, and 5 h against different target materials using a tribometer (Nanovea T-50, see Fig. 1). The tribometer variables were fixed to a load of 20 N, a speed of 35 revolutions per minute and a straight back and forth motion. In practice, a 0° working angle could not be maintained across the entire surfaces because the flaking surfaces were not perfectly flat, presenting irregular features like undulations. The angle of incidence was therefore not 0° at some spots on the surface. In total 10,500 strokes were executed on each target material, corresponding to a duration of 5 h. Five target materials were used for abrasion in this way: antler, ivory, bone, spruce wood and beech wood.

The samples were cleaned before every documentation of use-wear. Because of the metallic nature of the clamp, a mild cleaning process without the use of acids was preferred. The samples were placed in individual plastic bags filled with a 10% neutral soap solution (Valconox, Luminox). These were then immersed into an ultrasonic bath for 15 min (Branson 5800, 40 kHz, 22 °C). Then the samples were rinsed with tap water and put in a distilled water bath for 5 min. Finally, samples were left to air-dry.

### Infrared spectroscopy

The mineralogical composition of the use-wear polish was analysed with reflectance infrared spectroscopy. For this, a Bruker Hyperion 3000 microscope, attached to a Bruker VERTEX 80v spectrometer was used for all samples except the one abraded against bone. For this sample an Agilent Cary 620 microscope was used (spectral acquisition for all samples between 1350 and 600 cm^−1^). The zone of spectral acquisition was restricted with the microscope’s diaphragm so that a ~ 100 µm x ~ 100 µm zone was analysed (for the bone and beech wood samples, windows were closer to ~ 100 µm x ~ 50 µm to exclude zone without polish from the measurements). One such measurement was made on a zone of use-wear polish, possibly at a spot where the entire 10 mm^2^ zone was filled with polish, and another reference measurement was made on a fracture surface on one of the sides of the slabs that had not been abraded. For spectral deconvolution, the wavenumber region between 815 and 700 cm^−1^ was fitted with two pseudo-Voigt functions centred around 779 cm^−1^ and 798 cm^−1^. Another broad Gaussian function, centred around 760 cm^−1^, was included to improve the overall fit (residual fitting errors were typically between 0.006 and 0.1).

### Laser scanning microscopy

The polished slabs were first observed with a laser scanning microscope (LSM) to document changes in micro roughness and surface modifications caused by the use-wear polish. LSM measurements were performed using a Keyence VK-X 100 and a 50 × objective. To obtain 3D surface models of the surfaces affected by use-wear polish, nine tiles were stitched together, producing 0.67 mm × 0.75 mm measuring images. These surface models were then corrected for surface inclination and 2D surface profiles were extracted from the 3D surface models at different zones in the images (using the Gwyddion software^32^, version 2.4, released 2017-08-15, http://gwyddion.net/). These analyses were conducted in collaboration with C. Berthold of the Competence Center Archaeometry—Baden-Württemberg (CCA-BW) at Tübingen University’s Department of Geosciences.

## Supplementary information

Supplementary Figures.

## References

[1] Dunmore CJ, Pateman B, Key A (2018). A citation network analysis of lithic microwear research. J. Archaeol. Sci..

[2] Stemp WJ, Watson AS, Evans AA (2015). Surface analysis of stone and bone tools. Surf. Topogr. Metrol. Prop..

[3] Grace R (1996). Use wear analysis: The state of the art. Archaeometry.

[4] Curwen EC (1930). Prehistoric flint sickles. Antiquity.

[5] Keeley LH (1980). Experimental Determination of Stone Tool Uses: A Microwear Analysis.

[6] Semenov SA (1964). Prehistoric Technology: An Experimental Study of the Oldest Tools and Artefacts from Traces of Manufacture and Wear.

[7] Spurrell FCJ (1892). Notes on early sickles. Archaeol. J..

[8] Ollé A, Vergès JM, Longo L, Skakun N (2008). SEM functional analysis and the mechanism of microwear formation. ‘Prehistoric Technology’ 40 Years Later: Functional Studies and the Russian Legacy, Vol. 1783.

[9] Unger-Hamilton R (1984). The formation of use-wear polish on flint: Beyond the “deposit versus abrasion” controversy. J. Archaeol. Sci..

[10] Del Bene TA, Hayden B (1979). Once upon a striation: Current models of striation and polish formation. Lithic Use-Wear Analysis.

[11] Kamminga J, Hayden B (1979). The nature of use-polish and abrasive smoothing on stone tools. Lithic Use-Wear Analysis.

[12] Masson A, Coqueugniot E, Roy S (1981). Silice et traces d’usage: le lustré des faucilles. Publications du musée des Confluences.

[13] Meeks ND, de Sieveking G, Tite MS, Cook J (1982). Gloss and use-wear traces on flint sickles and similar phenomena. J. Archaeol. Sci..

[14] Yamada S, Anderson PC, Beyries S, Otte M, Plisson H (1993). The formation process of use-wear polishes. Traces et fonction: Les gestes retrouvés, vol. 50.

[15] Fullagar RLK (1991). The role of silica in polish formation. J. Archaeol. Sci..

[16] Witthoft J (1967). Glazed polish on flint tools. Am. Antiq..

[17] Anderson PC (1980). A testimony of prehistoric tasks: Diagnostic residues on stone tool working edges. World Archaeol..

[18] Andersen HH, Whitlow HJ (1983). Wear traces and patination on Danish flint artefacts. Nucl. Instrum. Methods Phys. Res..

[19] Buckley P, Hargreaves N, Cooper S (2018). Nucleation of quartz under ambient conditions. Commun. Chem..

[20] Mansur-Franchomme ME (1983). Scanning electron microscopy of dry hide working tools: The role of abrasives and humidity in microwear polish formation. J. Archaeol. Sci..

[21] van Ginneken B, Stavridi M, Koenderink JJ (1998). Diffuse and specular reflectance from rough surfaces. Appl. Opt..

[22] Sala, R. Formes d’ús i criteris d’efectivitat en conjunts de mode 1 i mode 2: Anàlisis de les deformacions per ús dels instruments lítics del Plistocè inferior (TD6) i mitjà (TG11) de la Sierra d’Atapuerca. (Universitat Rovira i Virgili (Departament d’Història i Geografia), 1997).

[23] Yamada S (2000). Development of the Neolithic: Lithic Use-Wear Analysis of Major Tool Types in the Southern Levant.

[24] Plisson H (1983). De la conservation des micro-polis d’utilisation. Bulletin de la Société Préhistorique Française. Comptes Rendus des Séances Mensuelles Paris.

[25] Christensen M (1998). Insight into the usewear mechanism of archaeological flints by implantation of a marker ion and PIXE analysis of experimental tools. Nucl. Instrum. Methods Phys. Res. Sect. B.

[26] Šmit Ž (1998). Usewear-induced deposition on prehistoric flint tools. Nucl. Instrum. Methods Phys. Res. Sect. B.

[27] Šmit Ž, Grime GW, Petru S, Rajta I (1999). Microdistribution and composition of usewear polish on prehistoric stone tools. Nucl. Instrum. Methods Phys. Res. Sect. B.

[28] Evans A, Donahue R (2005). The elemental chemistry of lithic microwear: An experiment. J. Archaeol. Sci..

[29] Jones J, Segnit E (1975). Nomenclature and the structure of natural disordered (opaline) silica. Contrib. Miner. Petrol..

[30] Spitzer WG, Kleinman DA (1961). Infrared Lattice Bands of Quartz. Phys. Rev..

[31] Sato K (1965). Infrared Lattice Vibration Spectra of Crystalline Quartz. J. Phys. Soc. Jpn..

[32] Nečas D, Klapetek P (2012). Gwyddion: An open-source software for SPM data analysis. Cent. Eur. J. Phys..

[33] Ibáñez JJ, Anderson PC, González-Urquijo J, Gibaja J (2016). Cereal cultivation and domestication as shown by microtexture analysis of sickle gloss through confocal microscopy. J. Archaeol. Sci..

[34] Schmidt P, Fröhlich F (2011). Temperature dependent crystallographic transformations in chalcedony, SiO_2_, assessed in mid infrared spectroscopy. Spectrochim. Acta Part A Mol. Biomol. Spectrosc..

[35] Cayeux L (1929). Les roches sédimentaires de France: roches siliceuses.

