# Supplementary material for: The mineralogy and structure of use-wear polish on chert
Source: Sci Rep. 2020 Dec 9;10:21512. doi: 10.1038/s41598-020-78490-0 (PMC7725782; doi:10.1038/s41598-020-78490-0)
Supplement: Supplementary file 1 — Supplementary Figures. [file 41598_2020_78490_MOESM1_ESM.pdf]

Supplementary online material for: **The mineralogy and structure of use-wear polish on chert**

Patrick Schmidt[1,2], Alice Rodriguez[3], Kaushik Yanamandra[4], Rakesh Kumar Behera[4], Radu Iovita[3,1]

[1] Department of Early Prehistory and Quaternary Ecology, Eberhard Karls University of Tübingen, Germany.
[2] Applied Mineralogy, Department of Geosciences, Eberhard Karls University of Tübingen, Germany.
[3] Anthrotopography Laboratory, Center for the Study of Human Origins, Department of Anthropology, New York University, New York, USA.
[4] Composite Materials and Mechanics Laboratory, Mechanical and Aerospace Engineering Department, New York University, Tandon School of Engineering, Brooklyn, New York, USA

Corresponding authors:
Patrick Schmidt (Orcid: 0000-0002-8727-6127)
Mail: patrick.schmidt@uni-tuebingen.de
Tel: +49 7071 29 74390
Radu Iovita (ORCID: 0000-0001-9531-1159)
Mail: iovita@nyu.edu

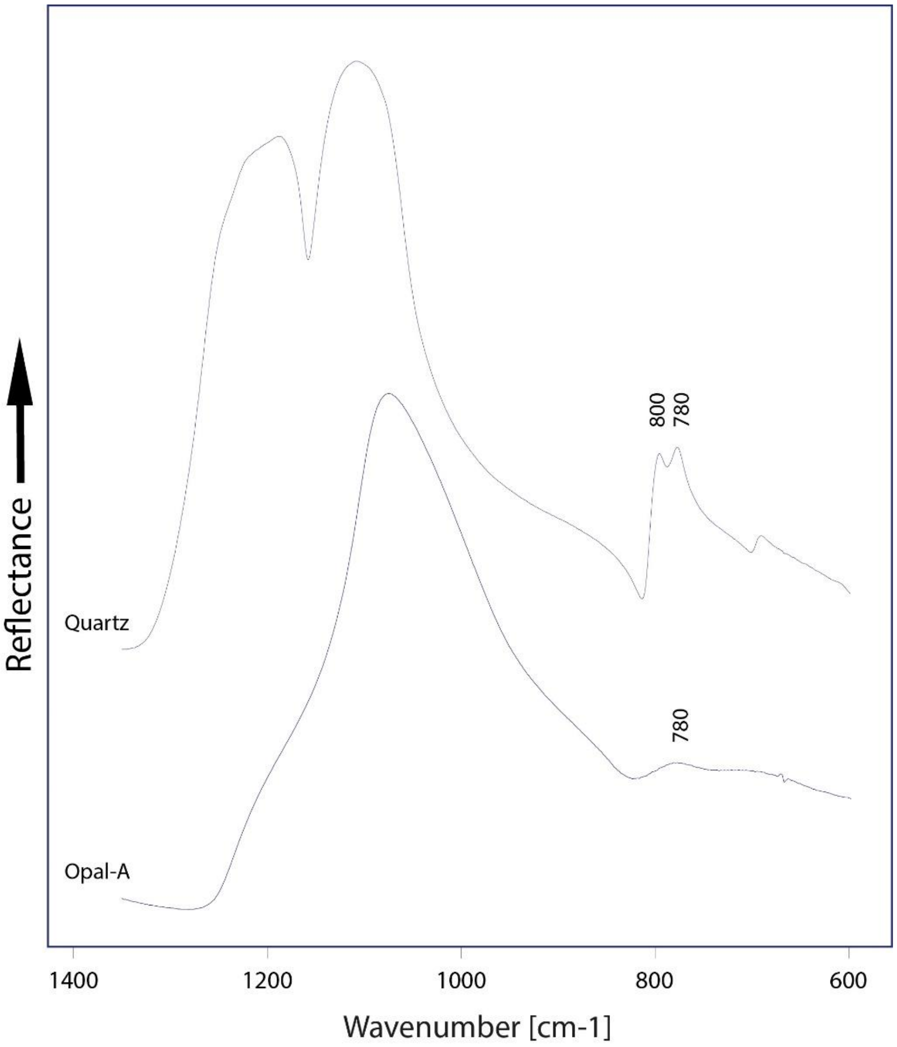

Fig. S1. Comparison of the unpolarised infrared reflectance spectrum for microcrystalline quartz in chert (top) and amorphous silica in opal-A (bottom). Note that the split of the B band into two components in quartz.

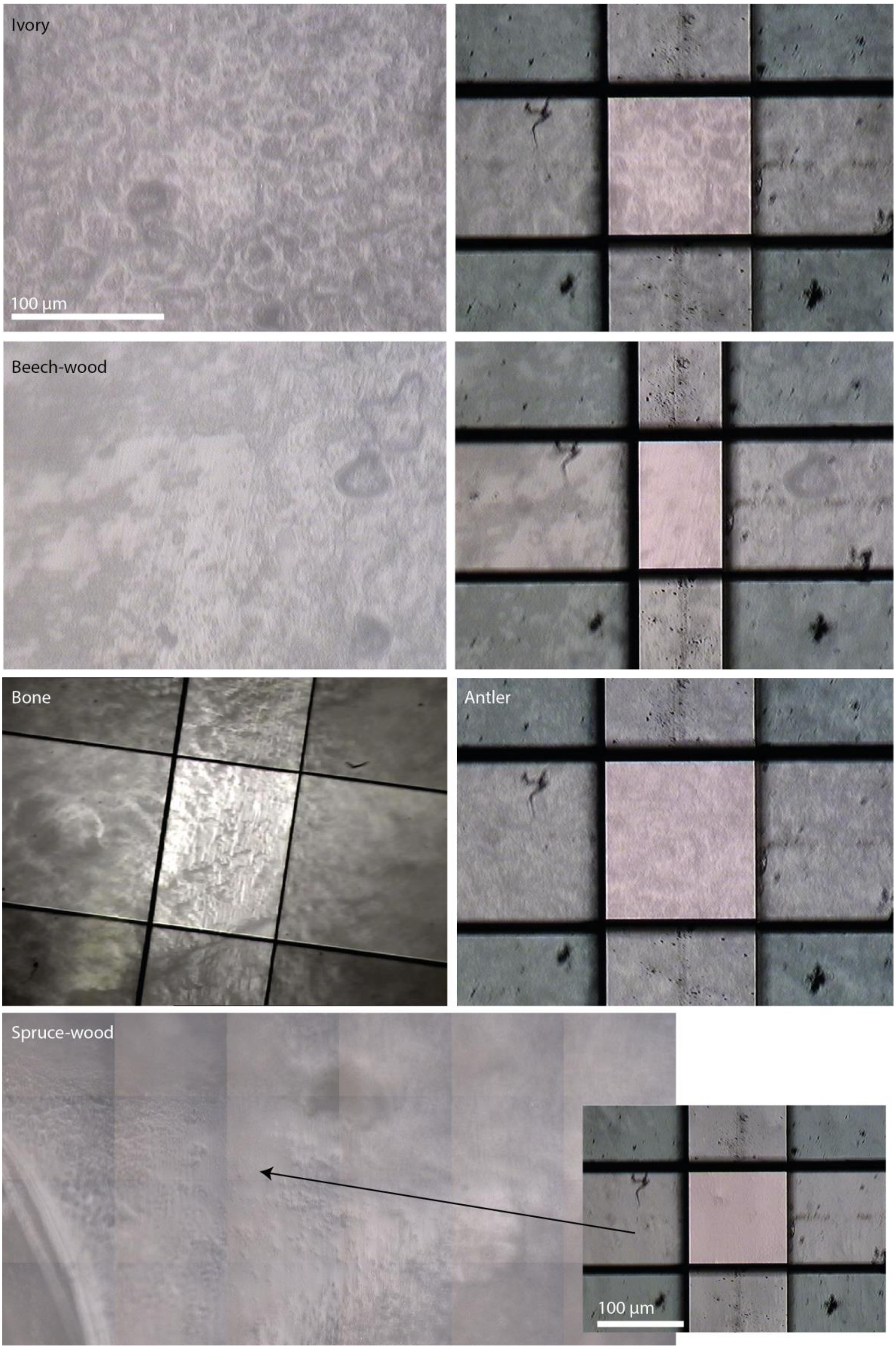

Fig. S2. Measured zones that produced the spectra of use-ware polish shown in the main text. The scale bar in the upper left photo is valid for all other photos, except the spruce wood measurement shown in the bottom.

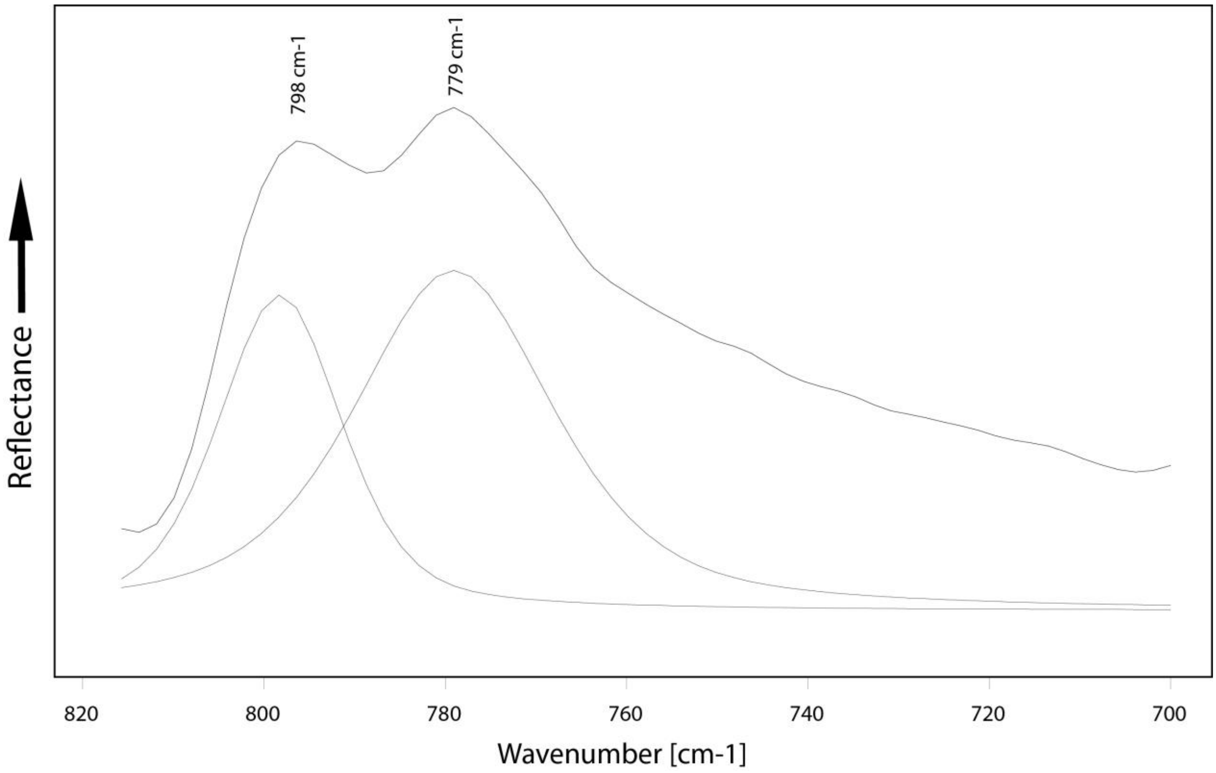

Fig. S3. Band components obtained by fitting the spectral zone between 815 cm^{-1} and 700 cm^{-1}. Upper line: measured spectrum. Lower lines: band components obtained by fitting. Note that both components are pseudo-Voigt functions.

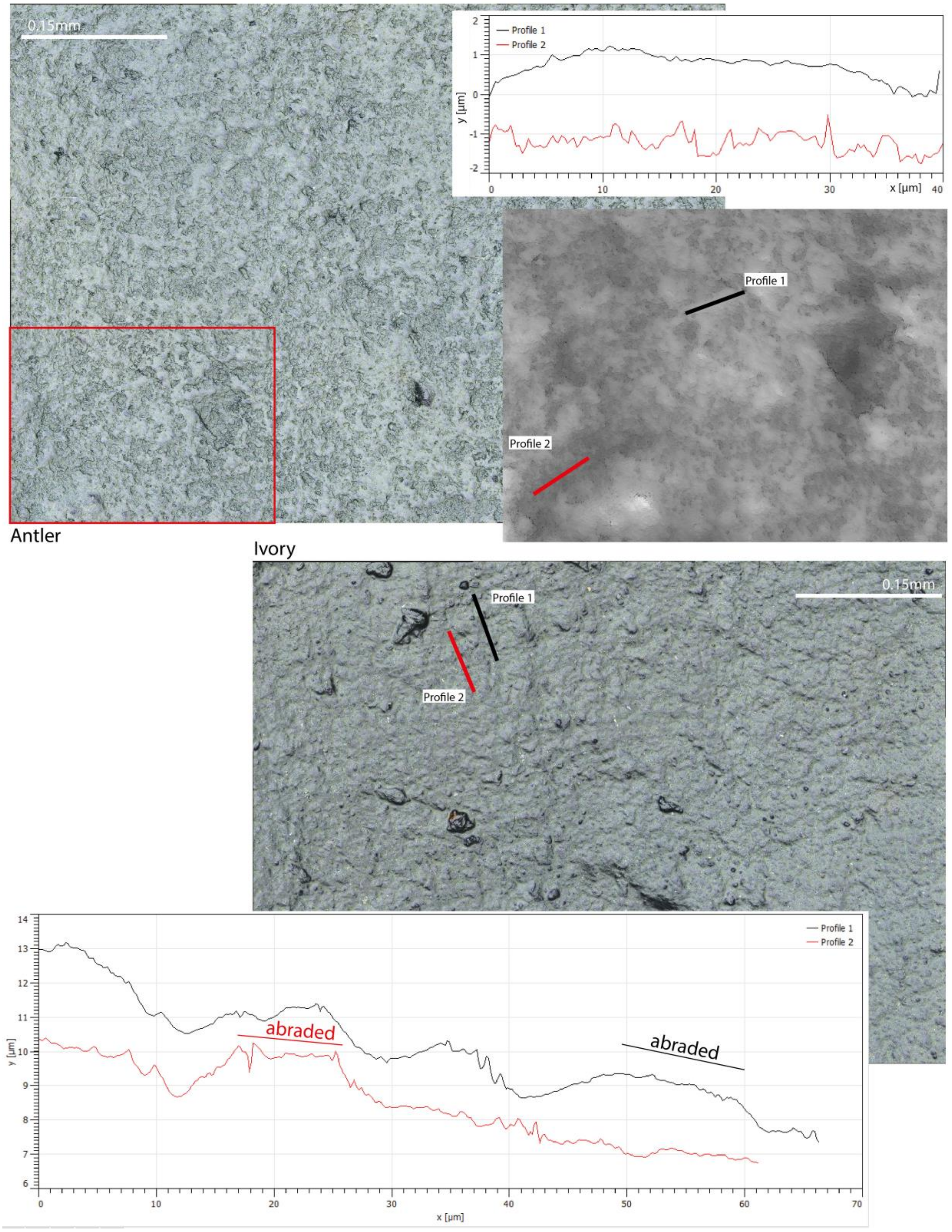

Fig. S4. 3D surface models of chert abraded against antler (top) and ivory (bottom). Note that in the antler sample, the zones of polish lie higher and are smoother than unpolished zones. For ivory polish, it can be seen that mainly the higher relief is abraded while the valleys are left untransformed.

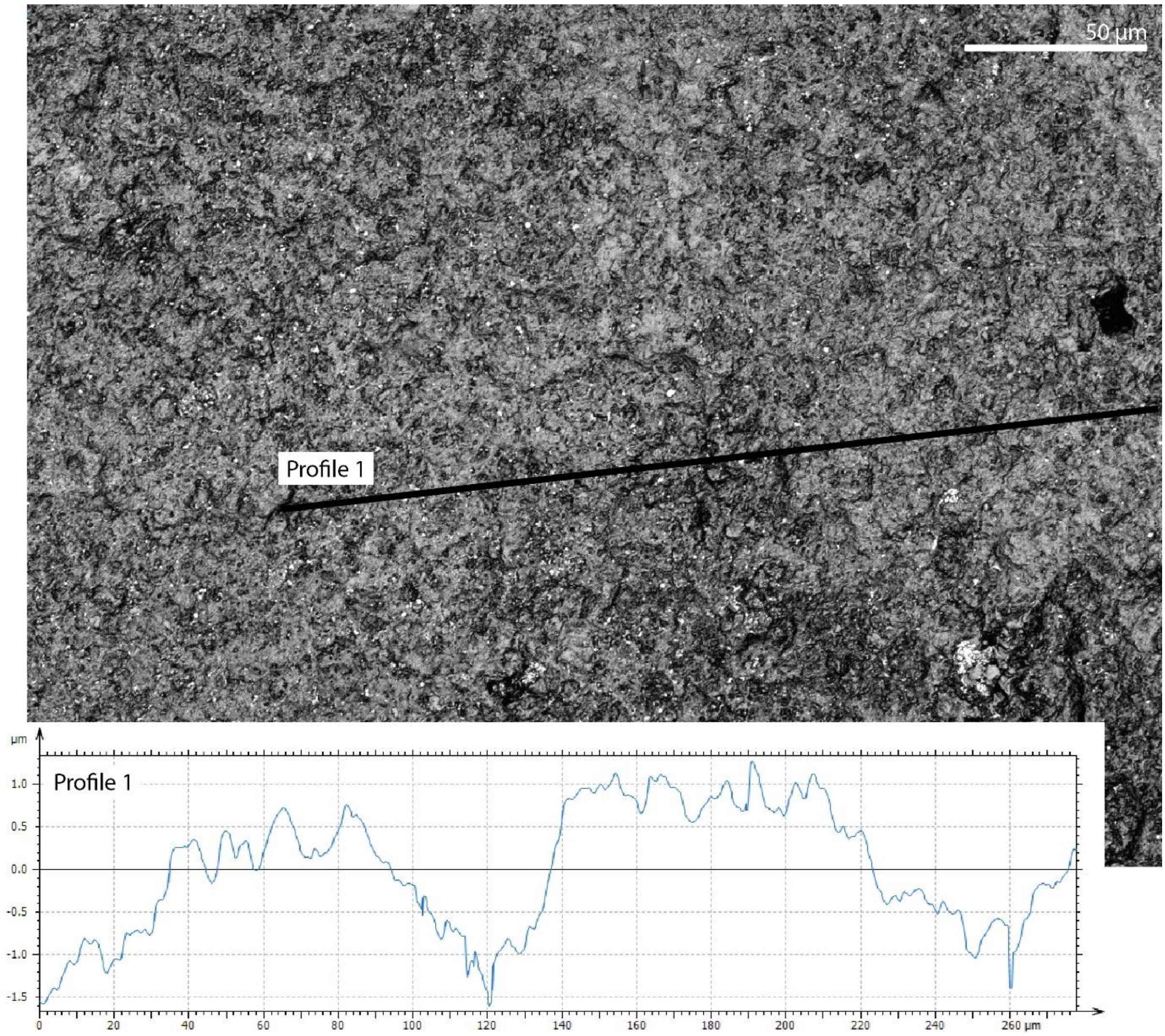

Fig. S5. 3D surface model of chert abraded against bone. While the same trend of flattened out higher lying parts is observable in this sample, the lower signal to noise ratio partially masks this effect.
